# Physicochemical characteristics of nanomaterials that affect pulmonary inflammation

**DOI:** 10.1186/1743-8977-11-18

**Published:** 2014-04-11

**Authors:** Hedwig M Braakhuis, Margriet VDZ Park, Ilse Gosens, Wim H De Jong, Flemming R Cassee

**Affiliations:** 1 National Institute for Public Health and the Environment (RIVM), PO Box 1, Bilthoven 3720BA, The Netherlands; 2 Department of Toxicogenomics, Maastricht University, PO Box 616, Maastricht 6200MD, The Netherlands; 3 Institute of Risk Assessment Sciences, Utrecht University, PO Box 80.163, Utrecht 3508TD, The Netherlands

**Keywords:** Nanoparticles, Inhalation exposure, Pulmonary toxicity, Particle characteristics, Surface reactivity, Risk assessment

## Abstract

The increasing manufacture and use of products based on nanotechnology raises concerns for both workers and consumers. Various studies report induction of pulmonary inflammation after inhalation exposure to nanoparticles, which can vary in aspects such as size, shape, charge, crystallinity, chemical composition, and dissolution rate. Each of these aspects can affect their toxicity, although it is largely unknown to what extent. The aim of the current review is to analyse published data on inhalation of nanoparticles to identify and evaluate the contribution of their physicochemical characteristics to the onset and development of pulmonary inflammation. Many physicochemical characteristics of nanoparticles affect their lung deposition, clearance, and pulmonary response that, in combination, ultimately determine whether pulmonary inflammation will occur and to what extent. Lung deposition is mainly determined by the physical properties of the aerosol (size, density, shape, hygroscopicity) in relation to airflow and the anatomy of the respiratory system, whereas clearance and translocation of nanoparticles are mainly determined by their geometry and surface characteristics. Besides size and chemical composition, other physicochemical characteristics influence the induction of pulmonary inflammation after inhalation. As some nanoparticles dissolve, they can release toxic ions that can damage the lung tissue, making dissolution rate an important characteristic that affects lung inflammation. Fibre-shaped materials are more toxic to the lungs compared to spherical shaped nanoparticles of the same chemical composition. In general, cationic nanoparticles are more cytotoxic than neutral or anionic nanoparticles. Finally, surface reactivity correlates well with observed pulmonary inflammation. With all these characteristics affecting different stages of the events leading to pulmonary inflammation, no unifying dose metric could be identified to describe pulmonary inflammation for all nanomaterials, although surface reactivity might be a useful measure. To determine the extent to which the various characteristics influence the induction of pulmonary inflammation, the effect of these characteristics on lung deposition, clearance, and pulmonary response should be systematically evaluated. The results can then be used to facilitate risk assessment by categorizing nanoparticles according to their characteristics.

## Introduction

In recent years, a large number of nanotechnology-enabled products have entered the global marketplace. In March 2011, the Nanotechnology Consumer Products Inventory contained 1317 products or product lines from over 30 countries, a growth of nearly 521% (from 212 to 1317 products) since the Inventory was first released in March 2006 [1]. Exposure to nanomaterials is on the rise, and because of uncertainty regarding their toxic characteristics, concerns have arisen that such materials pose new health risks for consumers, workers, and the environment.

An adequate risk assessment of nanomaterials requires information on both the exposure and hazard of their component particles. Inhalation is considered to be an important route of exposure to nanoparticles [2,3], especially in occupational settings. Many products, such as sprays, may likewise lead to inhalation by consumers [4,5]. With regard to hazard, numerous *in vitro* and *in vivo* studies have been conducted to determine whether inhalation of nanoparticles causes adverse effects. The most reported effect is pulmonary inflammation, largely indicated by an influx of neutrophils that can be observed in the bronchoalveolar lavage fluid *in vivo* and the induction of inflammatory cytokines in *in vitro* lung models eg. [6-13].

Nanomaterials are composed of primary and agglomerated particles that can vary in size, shape, charge, crystallinity, chemical composition and other characteristics, and this variety will increase even further in the future [14]. All these characteristics have been suggested to affect the toxicity of nanomaterials, but not all existing and emerging types of nanomaterials can be tested separately in studies to evaluate their safety. The current review therefore seeks to identify trends regarding their characteristics and pulmonary inflammation, as a key hazard indicator, by analysing published data on inhalation of nanoparticles. Ideally, this includes in depth analysis of characteristics that influence the mechanism underlying pulmonary inflammation e.g. that affect chemotactic signalling. Unfortunately, little information exists to elucidate the role of specific particle properties on details of the mechanism such as chemotactic signals. For this reason, we have limited our analysis to more generally reported effects of pulmonary inflammation and phagocytosis at a larger scale. The exposure assessment of nanomaterials, which is of major importance for the risk assessment of nanomaterials, is out of the scope of this review. As the induction of pulmonary inflammation results from a combination of their deposition, clearance, and interactions in the lungs, the characteristics influencing one or more of these processes will be discussed using data of peer reviewed papers. Ultimately, these results can be used to design safer nanomaterials and to identify those that need to be investigated further in terms of their health risks. For the risk assessment of nanomaterials, knowledge on toxicity-determining characteristics will help to categorise nanomaterials into hazard groups according to these characteristics.

Comparison across studies is often difficult due to the use of different experimental protocols and choice of endpoints, which largely influences the results. Therefore, our approach was to focus on investigations of multiple nanoparticles differing in one physicochemical characteristic within the same *in vivo* study. These studies are summarised in Table 1. We are aware of the fact that some studies use rather high exposures. Since there is no scientific consensus on when exposures are no longer realistic, and for the sake of including as much information as possible, we did include these studies in our review. In addition, we included both inhalation and intratracheal instillation studies. Since the dose rate together with the clearance rate will be the main driver for the retained dose, intratracheal instillation may lead to different effects than when using inhalation of aerosols; when available, the retained doses in the lungs are included.

**Table 1:** Inhalation studies investigating the effect of nanomaterial characteristics on lung deposition, clearance, and/or pulmonary inflammation

**Nanoparticle characteristic studied**	**Reference**	**Chemical composition**	**Primary particle size**	**Agglomerate particle size in air**	**Exposure time and type**	**Lung deposition, clearance, and translocation**	**Lung inflammation**
**Agglomerate size**	Ho et al. 2011 [15]	Zinc oxide	Not reported	35 nm CMD^1^	6 hours inhalation		Dose-dependent pulmonary inflammation. Exposure concentration: 2.4, 3.7, 12.1 mg/m^3^ for the 35 nm particles and 7.2, 11.5, 45.2 mg/m^3^ for the 250 nm particles.
				250 nm CMD			
**Agglomerate size**	Kreyling et al. 2002 [16]	Radio-labelled Iridium	Not reported	15 nm CMD	1 hour inhalation: 0.6 μg 15 nm; 6.0 μg 80 nm	Larger deposited fraction of 15 nm compared to 80 nm particles. Similar clearance kinetics via gastro-intestinal tract. Translocation very low, but higher for the 15 nm compared to the 80 nm particles.	
				80 nm CMD			
**Agglomerate size**	Kreyling et al. 2009 [17]	Radio-labelled Iridium	2 – 4 nm	20 nm CMD	1 hour inhalation: 0.6 μg 15 nm; 6.0 μg 80 nm	Translocation of 20 nm Iridium particles is larger compared to 80 nm Iridium particles	
				80 nm CMD		Translocation of Iridium particles is higher compared to similar sized carbon particles.	
**Chemical composition**							
		Iridium-labelled Carbon	5 – 10 nm	25 nm CMD			
**Agglomerate size**	Noël et al. 2012 [18]	Titanium dioxide	5 nm	30 and 185 nm agglomerates (2 mg/m^3^) 31 and 194 nm agglomerates (7 mg/m^3^)	6 hours inhalation: 2 mg/m^3^ and 7 mg/m^3^	Similar lung deposition of small and large agglomerates.	Exposure to both small and large agglomerates at 7 mg/m^3^ resulted in adverse effects. Exposure to the large agglomerates results in a significant increase in neutrophils in the lungs, while the small agglomerates did not.
**Agglomerate size**	Oberdörster et al. 2000 [10]	Platinum	Not reported	18 nm CMD	6 hours inhalation: 100 μg/m^3^ platinum and carbon; 40 μg/m^3^ Teflon	Ultra-fine particles all reach interstitial sites after translocation.	
		Carbon		26 nm CMD			
		Teflon		18 nm CMD			
**Agglomerate size**	Oberdörster et al. 2000 [10]	Teflon	Not reported	Starting with 18 nm CMD, size increasing over time	6 hours inhalation: ~50 μg/m^3^		Particles increased in size over time while particle number decreased; only freshly generated fumes (<100 nm) caused inflammation.
**Charge and solubility**	Cho et al. 2012 [19]	Silver	91.9 nm	Not applicable	Intratracheal instillation: 150 cm^2^/rat		Instillation of aluminum oxide, both cerium dioxides, cobalt oxide, both cupper oxides, nickel oxide, and both zinc oxides induced significant pulmonary inflammation, whereas instillation of the other nanoparticles did not.
		Aluminum oxide	6.3 nm				
		Cerium dioxide	9.7 and 4.4 nm				
		Cobalt oxide	18.4 nm				
		Chromium oxide	205 nm				
		Copper oxide	23.1 and 14.2 nm				Regarding the high-solubility nanoparticles, the inflammogenicity of copper oxide and zinc oxide was derived from their soluble ions. Other parameters showed a poor correlation with inflammation potential of nanoparticles.
		Magnesium oxide	15 nm				
		Nickel oxide	5.3 nm				
		Silicon dioxide	6.2 nm				
		Titanium dioxide	5.6 and 30.5 nm				
		Zinc oxide	10.7 and 137 nm				
**Charge**	Choi et al. 2010 [20]	Quantum dots (Zwitterionic, polar, anionic, cationic)	5 – 38 nm	Not applicable	Intratracheal instillation	A size threshold of ~34 nm determines whether there is rapid translocation of nanoparticles. Below 34 nm, surface charge is a major factor influencing translocation, with zwitterionic, anionic and polar surfaces being permissive and cationic surfaces being restrictive.	
		Silica (Polar)	56 – 320 nm				
		Polystyrene(Zwitterionic, polar, anionic)	7 – 270 nm				
**Chemical composition**	Heinrich et al. 1995 [21]	Diesel exhaust	-	0.25 μm MMAD^2^	2 year inhalation (rats)	Deposition, retention and total lung burden of diesel exhaust particles was highest compared to carbon black and titanium dioxide. Clearance was reduced in all groups; mostly reduced in group exposed to highest concentration of diesel exhaust.	Similar effects in all particle groups; carbon black induced the most lung tumours. Exposure concentration: 0.8, 2.5, 4.5, 7 mg/m^3^ diesel exhaust, 11.6 mg/m^3^ carbon black and 10 mg/m^3^ titanium dioxide.
		Carbon black	14 nm	0.64 μm MMAD	1 year inhalation (mice)		
		Titanium dioxide	15 – 40 nm	0.80 μm MMAD			
**Chemical composition**	Landsiedel et al. 2010 [22]	Titanium dioxide	40 nm (A)	-	5 days inhalation: 2, 10, 50 mg/m^3^ TiO_2_ (B); 0.5, 2.5, 10 mg/m^3^ ZrO_2_, CeO, SiO_2_, ZnO, CB; 0.1, 0.5, 2.5 mg/m^3^ MWCNT	Similar deposition of the particles. Only exposure to anatase titanium dioxide (B) resulted in particle overload in the lungs.	Titanium dioxide, cerium oxide, zinc oxide and MWCNT induced dose-dependent pulmonary inflammation. The effects of MWCNT were most severe and progressive. Zirconium dioxide, silicon dioxide and carbon black did not induce inflammation.
		Titanium dioxide	25 nm (B)	0.9 μm MMAD			
		Zirconium dioxide	40 nm	1.5 μm MMAD			
		Cerium oxide	40 nm	0.8 μm MMAD			
		Zinc oxide	60 nm	0.9 μm MMAD			
		Silicon dioxide	15 nm	1.2 μm MMAD			
		Carbon black	27 nm	0.8 μm MMAD			
		MWCNT	-	1.5 μm MMAD			
**Chemical composition**	Wang et al. 2010 [23]	Iron oxide	30 nm	Not reported	Spraying in the nose, twice daily for 3 days: 8.5 mg/kg bw Fe_2_O_3_ and 2.5 mg/kg bw ZnO	12 hours after exposure, zinc was detected in liver; 36 hours after exposure, iron was detected in liver and zinc in the kidneys.	Zinc oxide particles caused more severe changes in the liver while iron oxide caused more severe lung lesions.
		Zinc oxide	20 nm				
**Hydrophobicity**	Arts et al. 2007 [24]	Pyrogenic silica	Not reported	2 – 3 μm MMAD	5 days inhalation: 1, 5 and 25 mg/m^3^		Pyrogenic silica induced the most pronounced pulmonary inflammation compared to the other silica types.
		Silica gel					
		Precipitated silica					
**Hydrophobicity**	Reuzel et al. 1991 [25]	Hydrophilic silica	12 nm	1 – 120 μm MMAD	13 weeks inhalation: 1, 6, and 30 mg/m^3^	The 12 nm hydrophilic silica particles were more quickly cleared from the lungs compared to the other silica types.	Hydrophilic 12 nm (pyrogenic) silica induced more pulmonary inflammation compared to the other silica’s.
		Hydrophobic silica	12 nm				
		Hydrophilic silica	18 nm				
**Primary particle size**	Balasubramanian et al. 2013 [26]	Gold	7 nm	45.6 CMD	15 days inhalation: 0.086 -0.9 mg/m^3^ 7 nm; 0.053 – 0.57 mg/m^3^ 20 nm	7 nm gold NPs deposited in the brain, blood, small intestine and pancreas at greater mass concentration compared to 20 nm gold NPs. Clearance of the 20 nm particles is more effective compared to the 7 nm particles.	
			20 nm	41.7 CMD			
**Primary particle size**	Geraets et al. 2012 [27]	Cerium oxide	5 – 10 nm	1.02 μm MMAD	28 days inhalation: 11 mg/m^3^ 5–10 nm; 20 mg/m^3^ 40 nm; 55 mg/m^3^ < 5000 nm	Similar deposition in all groups; slow clearance in all groups; even slower clearance in 5 – 10 nm group. Very low translocation to secondary organs.	
			40 nm	1.17 μm MMAD			
			<5000 nm	1.4 μm MMAD			
**Primary particle size**	Gosens et al. 2010 [28]	Gold	50 nm	200 nm agglomerated	Intratracheal instillation: 1.6 mg/kg bw		Mild pulmonary inflammation; more effects for single 250 nm particles than for single 50 nm particles.
			250 nm	770 nm agglomerated			
**Primary particle size**	Gosens et al. 2013 [29]	Cerium oxide	5 – 10 nm	1.02 μm MMAD	28 days inhalation: 11 mg/m^3^ 5–10 nm; 20 mg/m^3^ 40 nm; 55 mg/m^3^ < 5000 nm		All materials induced dose-dependent pulmonary inflammation to the same extent.
			40 nm	1.17 μm MMAD			
			<5000 nm	1.4 μm MMAD			
**Primary particle size**	Horie et al. 2012 [30]	Nickel oxide	100 nm	Not applicable	Intratracheal instillation: 0.2 mg/0.4 ml		Nano-sized nickel particles induced inflammation and oxidative stress, while larger sized particles did not.
			600 – 1400 nm				
**Chemical composition**		Titanium dioxide	7 nm				
							Nano-sized nickel particles induced inflammation and oxidative stress, while the titanium dioxide particles did not.
			200 nm				
**Primary particle size**	Kobayashi et al. 2009 [31]	Titanium dioxide	4.9 nm	Not applicable	Intratracheal instillation: 1.5 mg/kg		Smaller particles induced greater inflammatory response at the same mass dose.
			23.4 nm				
			154.2 nm				
**Primary particle size**	Oberdörster et al. 1994 [32]	Titanium dioxide	20 nm	0.71 μm MMAD	12 weeks inhalation: 24 mg/m^3^ 20 nm TiO_2_; 22 mg/m^3^ 250 nm TiO_2_	Similar deposition in both groups. After deposition, disaggregation into smaller agglomerates. Retention halftime for 20 nm particles is longer compared to 250 nm particles.	
			250 nm	0.78 μm MMAD			
**Primary particle size**	Oberdörster et al. 2000 [10]	Platinum	Not reported	13 nm CMD	6 hours inhalation: ~110 μg/m^3^	Uptake of ultra-fine particles by lung macrophages was lower compared to larger sized particles.	
**Primary particle size**	Oberdörster et al. 2000 [10]	Titanium dioxide	20 nm	Not applicable	Intratracheal instillation	Both in rats and mice, 20 nm particles induced inflammation at lower mass dose compared to 250 nm particles. Exposure concentrations for the 20 nm particles: 31, 125, 500 μg in rats and 6, 25, 100 μg in mice. Exposure concentrations for the 250 nm particles: 125, 500, 2000 μg in rats and 25, 100, 400 μg in mice.	
			250 nm				
**Primary particle size**	Pauluhn et al. 2009 [13]	Aluminum oxyhydroxide	10 nm	1.7 μm MMAD	4 weeks inhalation: 0.4, 3 and 28 mg/m^3^	Translocation of 40 nm particles was higher compared to the 10 nm particles.	Both particles induced pulmonary inflammation to the same extent.
			40 nm	0.6 μm MMAD			
**Primary particle size**	Roursgaard et al. 2010 [33]	Quarts	100 nm	Not applicable	Intratracheal instillation: 50 μg		Both particles induced pulmonary inflammation to the same extent.
			1.6 μm				
**Primary particle size**	Sadauskas et al. 2009 [34]	Gold	2 nm (12 μg/ml)	Not applicable	5 intratracheal instillations within 3 weeks: 50 μl	Gold particles of all sizes detected in alveolar macrophages; translocation very low, but seems higher for 2 nm particles compared to larger sized particles.	
			40 nm (58 μg/ml)				
			100 nm (60 μg/ml)				
**Primary particle size**	Sayes et al. 2010 [35]	Silica	Not reported	37 nm CMD	1 or 3 day inhalation: 1.8 and 86 mg/m^3^		No induction of pulmonary inflammation.
				83 nm CMD			
**Primary particle size**	Stoeger et al. 2006 [36]	Carbonaceous nanoparticles	Six particles ranging from 10 – 50 nm	Not applicable	Intratracheal instillation: 5, 20 and 50 μg		Dose-dependent pulmonary inflammation; smaller nanoparticles induced more severe effects compared to larger nanoparticles.
**Primary particle size**	Zhu et al. 2008 [37]	Ferric oxide	22 nm	Not applicable	Intratracheal instillation: 0.8 and 20 mg/kg bw		Both particles induced pulmonary inflammation and oxidative stress to the same extent.
			280 nm				
**Shape**	Porter et al. 2012 [38]	Titanium dioxide spheres (anatase)	<70 – 200 nm	Not applicable	Pharyngeal aspiration: 15, 30 μg spheres; 7.5, 15, 30 μg nanobelts of 3 μm; 1.88, 7.5, 15, 30 μg nanobelts of 9 μm	Similar deposition for different shaped particles. Lung burden after exposure to nano-spheres was significantly lower compared to exposure to long nano-belts 112 days after exposure: impaired clearance of nano-belts.	Dose-dependent pulmonary inflammation in the animals exposed to titanium dioxide nano-belts. The longer nano-belts caused more severe pulmonary inflammation compared to the shorter ones. Shape and length affect pulmonary responses.
		Titanium dioxide nano-belts (anatase)	Length:3 μm (1 – 5 μm), width: 70 nm (40 – 120 nm) Length: 9 μm (4 – 12 μm), width: 110 nm (60 – 140)				
**Shape**	Schinwald et al. 2012 [39]	Silver nanowires	3 μm length, 115 nm diameter		Pharyngeal aspiration: 10.7, 17.9, 35.7, and 50 μg for 3, 5, 10 and 14 μm fibres, respectively	Length dependent restriction of macrophage locomotion. Fibre-length ≥ 5 μm resulted in impaired motility.	Length dependent inflammatory response in the lungs with threshold at a fibre length of 14 μm. Shorter fibres elicited no significant inflammation.
			5 μm length, 118 nm diameter				
			10 μm length, 128 nm diameter				
			14 μm length, 121 nm diameter				
			28 μm length, 120 nm diameter				
**Shape**	Schinwald et al. 2012 [40]	Graphene platelets	5.6 μm projected area diameter		Pharyngeal aspiration and intrapleural instillation: 50 μg	Prolonged retention of graphene platelets in the pleural space.	Exposure to graphene nanoplatelets caused pulmonary inflammation, while exposure to carbon black did not.
		Carbon black	10 nm				
**Shape**	Ma-Hock et al. 2013 [41]	Multi-walled carbon nanotubes	15 nm, fiber-shape	0.5 μm CMD	5 days inhalation: 0.1, 0.5, and 2.5 mg/m^3^ MWCNT, 0.5, 2.5, and 10 mg/m^3^ graphene, nanoplatelets and CB	The lung deposition was calculated to be 0.03 mg/lung MWCNT, 0.3 mg/lung graphene, 0.2 mg/lung graphite nanoplatelets, and 0.4 mg/lung carbon black.	Pulmonary inflammation was induced after exposure to multi-walled carbon nanotubes at all concentrations, and exposure to graphene at 10 mg/m^3^. The other exposures did not induce pulmonary inflammation. The lung burden did not correlate to the observed toxicity.
		Graphene	Up to 10 μm, flake	0.6 μm CMD			
		Graphite nanoplatelets	Up to 30 μm, flake	0.4 μm CMD			
		Carbon black	50 – 100 nm	0.4 μm CMD			
**Solubility**	Cho et al. 2011 [42]	Zinc oxide	10.7 nm 137 nm	Not applicable	Intratracheal instillation: 50 and 150 cm^2^/rat		Zinc oxide particles caused severe pulmonary inflammation probably caused by zinc ions released from rapid dissolution of inside phagolysosomes.
		Nickel oxide	5.3 nm				
		Titanium dioxide	30.5 nm				
**Solubility**	Cho et al. 2012 [43]	Nickel oxide	10 – 20 nm	Not applicable	Intratracheal instillation: 30, 100, 300 cm^2^/ml NiO; 3, 10, 30 cm^2^/ml ZnO and CuO		Pulmonary inflammation is caused by nickel oxide nanoparticles and not the ions, zinc oxide and copper oxide nanoparticles caused particle-specific eosinophil recruitment. *In vitro*, zinc and copper ions caused the observed adverse effects.
		Zinc oxide	<10 nm				
		Copper oxide (and their aqueous extracts)	<50 nm				
**Surface reactivity**	Van Ravenzwaay et al. 2009 [44]	Titanium dioxide (70% anatase, 30% rutile)	20 – 30 nm	1.0 μm MMAD	5 days inhalation: 88 mg/m^3^ 20–30 nm TiO2; 274 mg/m^3^ 200 nm TiO2; 96 mg/m^3^ Quartz		Both titanium particles induced reversible effects, while the effects caused by quartz remained. Quartz induced the most prominent pulmonary inflammation while the surface area of deposition was the lowest.
**Chemical composition**							
		Titanium dioxide (rutile)	200 nm	1.1 μm MMAD			
		Quartz		1.2 μm MMAD			
**Surface reactivity**	Warheit et al. 2007 [11]	Nano-titanium	Not reported	140 nm	Intratracheal instillation: 1 and 5 mg/kw bw		Only the titanium dioxide particles with the highest surface reactivity induced pulmonary inflammation.
		Nano-titanium		130 nm			
		Fine titanium		380 nm (size in water)			
**Surface reactivity**	Warheit et al. 2007 [12]	Nano-Quartz	50 nm	Not applicable	Intratracheal instillation: 1 and 5 mg/kg bw		Pulmonary inflammation was not dependent on particle size but correlated well with the haemolytic potential of the particles.
		Nano-Quartz	12 nm				
		Fine Quartz	300 nm				

^1^CMD: count median diameter.

^2^MMAD: mass median aerodynamic diameter.

Studies are listed according to the nanoparticle characteristic studied, in alphabetical order.

### Deposition of nanoparticles in the lungs

**Figure 1:**
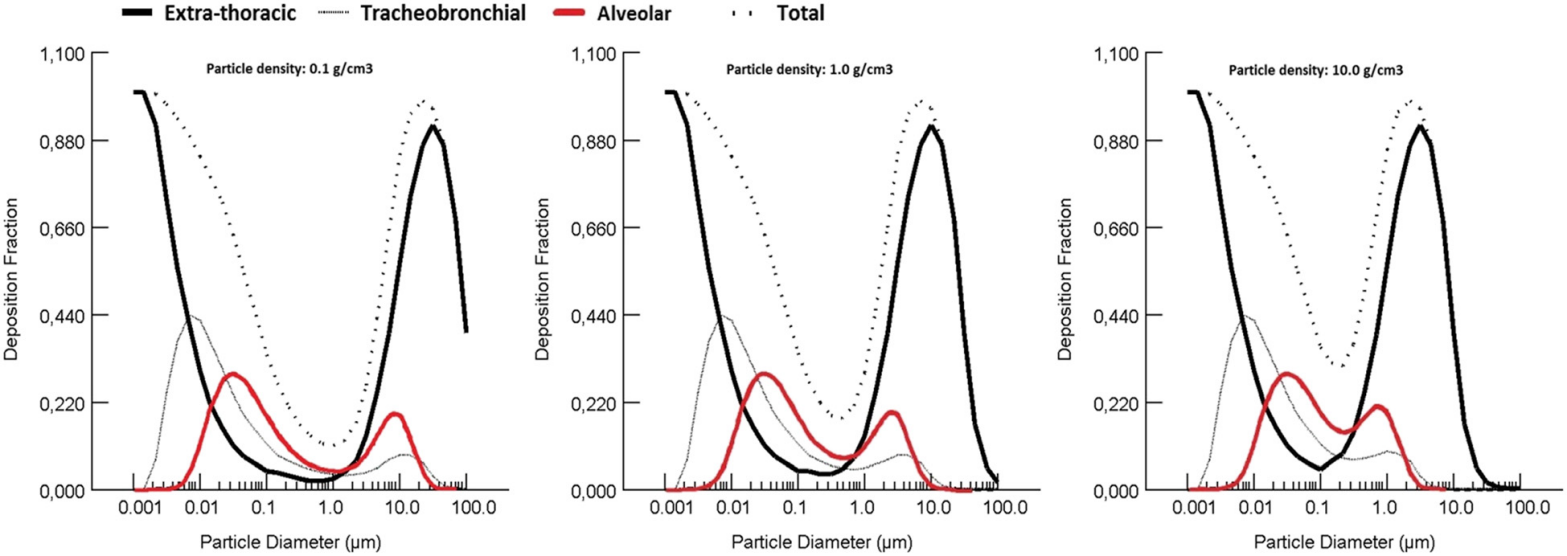
**Deposition of particles in different regions of the lung depends on particle size and density.** Particle size ranges from 1 nm to 100 μm, particle density tested: 0.1 g/cm^3^ (left panel), 1.0 g/cm^3^ (centre panel) and 10.0 g/cm^3^ (right panel) (Simulation made in Multiple Pathway Particle Dosimetry Model V2.1 Copyright ARA 2009, based on human oronasal-normal augmenter breathing). The figure shows the deposition of inhaled particles in the extra-thoracic region (black line), the tracheobronchial region (grey line), and the alveolar region (red line). In the alveolar region, the deposition is the highest for nanoparticles with a primary or agglomerate particle size between 10 nm and 100 nm, regardless of the density. For particles with a primary or agglomerate size between 100 nm and 1 μm, the (agglomerate) density influences the deposition in the lungs: in this size range particles/agglomerates with a higher density will deposit more efficiently in the alveolar region compared to particles/agglomerates with a lower density.

A primary or individual nanoparticle (also called “ultrafine particle”) has a mean primary diameter of <100 nm, compared to >1 μm for a microparticle (also called “fine particle”). Primary particles tend to agglomerate, or aggregate, into larger particles. As they travel through the air from the point of generation to the point of exposure, the size of the primary and agglomerated particles determines their lung deposition pattern. (Figure 1) [45-47]. When nanoparticles are agglomerated in air, measurements of nanoparticle size will give the size of the agglomerates and not the primary particles [48]. The size of the agglomerates can be measured with different techniques, depending on the size of the agglomerates. Optical particle sizers (OPS) and aerodynamic particle sizers (APS) can measure the aerodynamic particle size ranging from 300 nm to 10 μm or 500 nm to 10 μm, respectively [49,50]. The aerodynamic particle size is mostly given as a mass median aerodynamic diameter (MMAD). Differential mobility analysers (DMA) and scanning mobility particle sizers (SMPS) can measure the electrical mobility diameter of particles ranging from 2.5 nm to 1 μm, depending on the type of SMPS used. The electrical mobility diameter is mostly given as a count median diameter (CMD). The aerodynamic size depends not only on the physical size of the particles but also on the density of the particles, while the electrical mobility size depends on the physical size alone [50].

Nanoparticles with a primary or agglomerate particle size between 10 and 100 nm will deposit more efficiently in the alveolar region compared to particles with an agglomerate particle size between 0.1 and 1 μm [45-47,51]. In the alveoli the airflow is minimal, therefore, for nanoparticles between 10 and 100 nm, the mechanism of deposition in the lungs is diffusion [52]. Several *in vivo* inhalation studies [10,16,17,21] show that particles of smaller agglomerate size deposit more efficiently in the alveolar region than those of larger agglomerate size. Particles that differ in primary particle size but have the same agglomerate diameter show similar deposition fractions [18,22,27,32]. As stated above, for agglomerated nanoparticles with an aerodynamic size above 300 nm, the density affects deposition. For these large agglomerates, increasing the density increases their deposition in the lungs, including in the alveolar region (Figure 1). It should be noted that the density of agglomerated particles is lower than the material density of the particles itself. Shape is also a factor. Primary and agglomerated nanoparticles occur in forms such as spheres, rods, fibres, wires, belts, triangles, and platelets. Shapes with a high aspect ratio, like fibres, have an aerodynamic size that is about three times their actual diameter; long fibres can deposit in the upper airways due to interception by touching the surface of the airways [53,54].

For particles to induce pulmonary inflammation, they must deposit in the alveolar region. When the agglomerate size of nanoparticles is <100 nm but above 10 nm, a considerable part of them will deposit in the alveolar region (about 30% of the particles) [45,46,55]. Although below 30 nm, the deposition shifts from the alveoli more towards the tracheobronchial region.

Using the above information, one can predict the dose of nanomaterials in the lung by using the Multiple Pathway Particle Dosimetry model (MPPD model). The model uses the morphology of the lung, respiratory conditions, and particle size (either CMD or MMAD), particle density, and exposure concentration to predict deposition in the various regions of the lung [51]. The exposure concentration determines the total amount of the nanoparticles that will deposit in the different regions of the lungs; it does not directly influence the deposition fraction in different regions of the lungs. It is important to note that the MPPD model gives an approximation of the deposition of particles in the lungs and results should be viewed with caution. For example, Figure 1 shows extra-thoracic deposition of 1 nm particles and no tracheobronchial or alveolar deposition in humans during oronasal breathing, while there is substantial deposition of 1 nm particles in the tracheobronchial region in humans during oral breathing (about 24%) [46]. In addition, nanoparticles can be polydispersed in the air, resulting in a range of agglomerate sizes within a cloud of nanoparticles. When selecting CMD in the MPPD model, the polydispersity of the particles is not taken into account and the resulting lung deposition pattern should be interpreted as an estimation with uncertainties.

Another complicating factor is that nanoparticle agglomerate size changes over time by coagulation in air. The process is predominantly determined by Brownian motion and depends on the concentration of particles as they travel from site of generation to site of exposure; it also depends on the time required to reach the exposure site [56]. Nanomaterials are generated with a certain primary particle size but then tend to agglomerate, resulting in a lower number of particles with an increasing agglomerate size. The higher the particle number at generation, the faster these agglomerates are formed: nanoparticles of 30 nm primary size at a number concentration of 10^7^ particles/cm^3^ are stable for a maximum of 10 seconds, while the same nanoparticles at a number concentration of 10^6^ particles/cm^3^ are stable for a maximum of 100 seconds [57]. With longer travel times, the agglomerates increase in size. The speed and extent of agglomeration also depend on nanoparticle characteristics like surface charge, type of coating, and hygroscopicity. Particles with the same surface charge repel each other, whereas neutral particles more easily agglomerate. Similar, coatings can cause nanoparticles to repel or attract. Hygroscopicity describes particle response to water molecules in the environment, depending on the relative humidity. For example, they may attract molecules and grow many times their original size at increasing relative humidity [58], or they may lose their own water content to evaporation, shrinking in size, at decreasing relative humidity.

When the influence of particle size is investigated, it should be measured as closely as possible to the site of exposure and not at the site of generation as a primary nanoparticle may constantly change its agglomerate size. After deposition of inhaled nanoparticles, agglomeration usually plays a minor role since the peripheral lung surface area is so large that the probability of two nanoparticles landing on each other is rather low for a diffusion-driven deposition. This is in contrast to deposition patterns of larger particles, which often congregate in ‘hot-spots’. The binding kinetics of proteins as influenced by nanoparticle charge and other physicochemical properties of the surface is the more important mechanism after deposition. In animal studies the lung deposition can be measured, while in humans the deposition of particles in the different regions of the lungs can be modelled by the MPPD model. The results of the MPPD model should be interpreted with caution, as they are an estimation of what happens in reality.

### Clearance of nanomaterials from the lungs

When nanoparticles are not exhaled, but deposited in the respiratory tract, there are several transport pathways to clear them. The sooner particles are cleared from the lungs, the smaller the likelihood that pulmonary inflammation will develop. The most prevalent mechanism for solid particle clearance in the alveolar region is mediated by alveolar macrophages, through phagocytosis [52]. Once the macrophages have taken up particles, they move gradually toward the mucociliary escalator and are subsequently swallowed and cleared from the body through the gastrointestinal tract.

The retention time of particles in the lung depends on the deposition site and the interaction of particles with the inner lung surface. Particles that deposit in the conducting airways have a short retention time due to efficient mucociliary and cough clearance. The retention time increases when particles deposit deeper in the lungs, given the increased pathway length and decreased mucous velocity [59]. For microparticles, the retention half-time in the alveolar region is about 70 days in rats and up to 700 days in humans [52]. For nanoparticles, the retention half-time tends to be longer because they can deposit in the alveolar region when their (agglomerate) size is between 10 nm and 100 nm. Even with a short retention time and complete phagocytosis by alveolar macrophages, pulmonary inflammation may still occur, as macrophages are well known for the release of pro-inflammatory mediators in response to the uptake of nanoparticles [60,61].

If not cleared by phagocytosis, nanoparticles can reach pulmonary interstitial sites from which they are transported to the local lymph nodes. In addition, translocation of particles into the blood circulation can occur by crossing the lung barrier in the alveolar region [62]. Subsequently, the particles are cleared from the body by the liver, gastrointestinal tract, or kidneys. However, translocated particles may be able to reach organs beyond the lung, where they can accumulate, and might cause damage. When the deposition of particles in the lung overwhelms the clearance mechanisms of the lung, this may result in a retained lung burden or accumulation of particles in the lung, which is greater than expected from linear kinetics. This situation is called lung particle overload [7,63-66]. In rats, particle overload may result in sustained inflammation, fibrosis and induction of lung tumours, but the evidence on whether this situation occurs in humans is inconclusive [67].

For all these lung clearance and transport pathways, several nanomaterial characteristics are of influence.

#### Particle size

Size-dependent differences are important in the cascade of events leading to effective macrophage-mediated clearance. After deposition, agglomerates can disagglomerate into the primary particles [26,32], or primary particles can agglomerate after contact with the lung lining fluid [68]. At the site of deposition, the lung is thus exposed to large agglomerates, smaller agglomerates, or primary particles, as can be verified by transmission electron microscopy (TEM). Microparticles or large nanoparticle agglomerates, with a particle/agglomerate size of >1 μm, are easy for macrophages to phagocytize [59], but single nanoparticles and small agglomerates are more difficult [69-71], and the smaller the nanoparticles, the less efficient their clearance [72]. Within 24 hours after exposure, alveolar macrophages phagocytize only 20% of nanoparticles compared to 80% of microparticles [52]. The nanoparticles that are not phagocytized are retained in the interstitium and in epithelial cells. Several *in vivo* inhalation studies report decreased clearance of nanoparticles from the lungs compared to larger-sized particles, resulting in increased retention time [32,69,70,73]. Increased retention gives nanoparticles the opportunity to translocate through the lung barrier. One study found low amounts (<0.2% of the inhaled dose) of cerium oxide in secondary organs [27]. Another study reported no difference in clearance rate between 15 nm and 80 nm radio-labelled iridium particles in rats. However, the translocation of the 15 nm particles was higher compared to the 80 nm particles. Particles were found in secondary organs at fractions of <0.002 and 0.001 of deposited dose of the smaller and larger particles, respectively [16]. This was confirmed in a follow-up study, in which translocation of 20 nm particles to secondary target organs was higher compared to 80 nm particles [17]. Another group found that translocation of gold nanoparticles of 2 nm, 40 nm, and 100 nm was very low, but greater for the 2 nm particles compared to the larger particles [34]. Similarly, after exposure to gold nanoparticles of 7 nm and 20 nm primary size (both having an agglomerate size of 45 nm), the 7 nm particles were more subject to translocation and more heavily distributed in secondary organs than the larger particles. However, the 20 nm particles were detected at higher levels in the aorta and faeces [26]. In contrast to these findings, when rats were exposed via inhalation to aluminum oxyhydroxide nanoparticles of 10 nm and 40 nm primary size (1.7 μm and 0.6 μm aerodynamic size), the translocation of the larger particles was higher compared to the smaller particles. The particles translocated to the lung-associated lymph nodes but were not detected in any other secondary organ [13]. The larger aerodynamic size of the 10 nm particles probably resulted in a lower deposited fraction in the alveoli compared to the 40 nm particles, which might explain the higher translocation rate of the 40 nm particles.

Overall, single nanoparticles and agglomerates of <100 nm are less efficiently phagocytized by alveolar macrophages compared to microparticles or large agglomerates of >1 μm [69,70,73]and less efficiently cleared by mucociliary clearance [71,72]. Increased retention of nanoparticles in the lung may damage the lungs or may result in the translocation of the nanoparticles to secondary organs. It is important to note that in general, clearance by translocation reported in the studies is very low, below 0.5% of the exposure concentration [16,17,27,34]. Over time, this may still accumulate to significant amounts for persistent nanoparticles, but no studies are available to demonstrate this.

#### Shape

Clearance from the lung is notably influenced by the shape of the particles [9]. Rigid fibres may more readily be entrapped in the lungs compared to spherical particles [74]. In rat inhalation studies, longer fibres appeared to be more difficult to clear than shorter ones [75]. Fibres longer than about 15–20 μm cannot be completely phagocytized by individual lung macrophages [76], resulting in frustrated phagocytosis in which adjacent cells attempt to phagocytize the same fibre [77]. In mice exposed to silver nanowires of different lengths via pharyngeal aspiration, shorter fibres with a length of 3, 5 and 10 μm could be completely phagocytized by alveolar macrophages whereas longer fibres with a length of 14 μm induced frustrated phagocytosis [39].

Exposure of mice to anatase titanium dioxide particles of various shapes (nanospheres, short nanobelts of 1 – 5 μm, and long nanobelts of 4 – 12 μm) resulted in a lung deposition of 135 μg both for the animals exposed to nanospheres and long nanobelts, but clearance was affected by particle shape. At 112 days after exposure, the lung burden was significantly lower in mice exposed to nanospheres (45 μg) than in those exposed to long nanobelts (60 μg) [38]. Nanoplatelets can also impair clearance compared to spherical nanoparticles. After intrapleural instillation in mice, graphene nanoplatelets induced prolonged retention in the pleural space whereas carbon black did not [40]. The study shows that the aerodynamic diameter of graphene nanoplatelets is much smaller than the projected area diameter, giving the platelets the opportunity to deposit in the alveoli [40]. In conclusion, long fibre-like or platelet nanoparticles are more readily entrapped in the lung and are more difficult to clear than shorter ones or spherical nanoparticles [38-40,76,77].

#### Chemical composition

Long-term exposure to diesel exhaust particles at concentrations of 0.8, 2.5, 4.5 and 7 mg/m^3^, carbon black at 11.6 mg/m^3^, and titanium dioxide (80% anatase, 20% rutile) at 10 mg/m^3^ resulted in impaired clearance from the lungs for all three types. After 18 months of exposure and a recovery period of 3 months, lung clearance was still impaired and had not returned to normal levels. At that time point, the retained masses of the test materials were 47.7 mg/lung, 45.2 mg/lung and 37.8 mg/lung for diesel exhaust, carbon black and titanium dioxide, respectively. The strongest effect on lung clearance was caused by exposure to the highest concentration of diesel exhaust particles compared to similar concentrations of titanium dioxide and carbon black [21]. The effects after short-term inhalation of 5 days were tested for two types of titanium dioxide (anatase or 80% anatase with 20% rutile), zirconium dioxide, cerium dioxide, zinc oxide, silicon dioxide, carbon black, and multi-walled carbon nanotubes [22]. All nanoparticles were tested at concentrations of 0.5, 2.5 and 10 mg/m^3^, except for titanium dioxide nanoparticles that were tested at 2, 10 and 50 mg/m^3^ and multi-walled carbon nanotubes that were tested at 0.1, 0.5 and 2.5 mg/m^3^. Only exposure to anatase titanium dioxide, at the highest concentration tested, resulted in reduced lung clearance: the retained dose was 1635 μg directly after 5 days inhalation and 1340 μg at day 21–29 [22]. For the other nanoparticles tested, the retained doses in the lungs were at least a factor 4 lower, which might be explained by the lower exposure concentration.

Regarding translocation, the accumulation of carbon-iridium particles in secondary organs was lower compared to similar-sized iridium particles after inhalation [17]. When rats were exposed to either 30 nm iron oxide particles (8.5 mg/kw bw) or 20 nm zinc oxide particles (2.5 mg/kg bw), there were differences in translocation rate and distribution in the body. After 12 hours of exposure, zinc was detected in the liver, and after 36 hours, iron was detected in the liver and zinc was detected in the kidneys [23]. These measurements made no distinction between particles and ions, but can likely be explained by differences in dissolution rates. Overall, clearance and translocation rates differ depending on the chemical composition of the nanoparticles.

#### Surface charge

Surface charge of nanoparticles may also influence their translocation rate. Endogenous proteins like albumin adsorb to the surface of charged nanoparticles, thereby increasing their hydrodynamic size, altering their surface charge [78,79], and decreasing their surface reactivity and translocation rate [20]. The higher the surface charge density, the more proteins are adsorbed [80]. On the other hand, zwitterionic or neutral organic coatings prevent adsorption of serum proteins [81]. Examples are zwitterionic cysteine and polar PEG ligands that lead to rapid translocation of nanoparticles to the mediastinal lymph nodes [20]. Overall, charged particles attract proteins and thereby reduce their translocation rate [20,80,81]. In addition, the attracted proteins can form a corona around the nanoparticles and alter their recognition and uptake by alveolar macrophages.

#### Dissolution in physiological media

Many nanoparticles are insoluble and retain their physical shape after deposition. Others, like zinc oxide, copper oxide, nickel oxide, iron oxide, silicon dioxide, and silver nanoparticles can dissolve at various rates. The epithelium of the respiratory tract is covered with a lining fluid, and materials that dissolve in this fluid are readily transferred to the blood [82]. Only a few studies focused on the relation between particle dissolution and clearance from the lungs. One study suggests that after intratracheal instillation, the ability of metals to translocate from the lungs into the systemic circulation appears to be related to their solubility in water [83]. Another study showed that for cobalt oxide particles, the *in vitro* intracellular particle dissolution rate in alveolar macrophages was similar to the *in vivo* transfer to the blood [82]. However, when the released ions precipitate and/or transform, they might not be readily transferred and remain in the lungs. Therefore, the correlation between intracellular particle dissolution and *in vivo* clearance by transfer to the blood is only valid for specific particles of which the intracellular dissolution rate is the limiting step in their clearance [82]. After deposition, some particles will be taken up by alveolar macrophages. Phagolysosomes inside the macrophages contain proteolytic enzymes, oxygen radicals, chelators, precipitators, and a low pH of about 5, all of which may affect the engulfed particles. The low pH will increase particle dissolution. If particles release ions that destabilize the membrane of the lysosome, lysosomal content can leak and result in cell death, releasing the ions from the macrophages into the lungs. By affecting the barrier function of the lung epithelium, ions may enable intact nanoparticles to enter the bloodstream as well [82,84].

### Pulmonary inflammation induced by nanomaterials

After deposition in the alveoli, nanoparticles interact with the alveolar epithelium. Nanoparticles can escape clearance by alveolar macrophages resulting in prolonged interaction with the alveolar epithelium [85]. At a high deposited dose of nanoparticles, there is epithelial injury, the immune system will treat the presence of the particles as a threat and inflammation ensues. As a response to the epithelial injury, there is an influx of neutrophils into the alveolar region [86-88]. The epithelial cells generate chemotactic factors that stimulate the migration of macrophages [89]. A prolonged exposure of epithelial cells to nanoparticles may result in hyper-secretion of chemo-attractants into the alveolar space. It is possible that this may disrupt the normal chemotactic gradient within the lung and result in particle-laden macrophages remaining within the respiratory region instead of migrating to the mucociliary escalator for clearance [90].

The effect of nanomaterials after inhalation that is reported most often is that of pulmonary inflammation, characterized by an influx of polymorphonuclear neutrophils, which may be transient or persistent [10-13,15,18,21-25,28-31,33,36-38,42-44]. At the cellular level, nanoparticle exposure can induce oxidative stress by the production of reactive oxygen species (ROS), which may be generated directly by particle structures in or near the cell or may arise more indirectly due to the effects of internalized particles on mitochondrial respiration [91] or the depletion of antioxidant species within the cell [92]. Oxidative stress can damage cells by peroxidising lipids, inducing inflammation, and altering proteins and DNA [93]. It can mediate a number of processes in the cells, such as apoptosis, DNA adduct formation, and pro-inflammatory gene expression [94]. All of these have been reported following exposure to some types and concentrations of nanoparticles [43,95-98]. Therefore, ROS production is considered the main underlying biochemical process in nanotoxicology, leading to inflammatory and other secondary processes that can ultimately cause cell damage and even cell death [93,99-101]. In the lung, persistent oxidative stress and inflammation after exposure to particulate matter are thought to cause fibrosis; in brain tissue, they are associated with neurodegenerative diseases [102,103].

**Figure 2:**
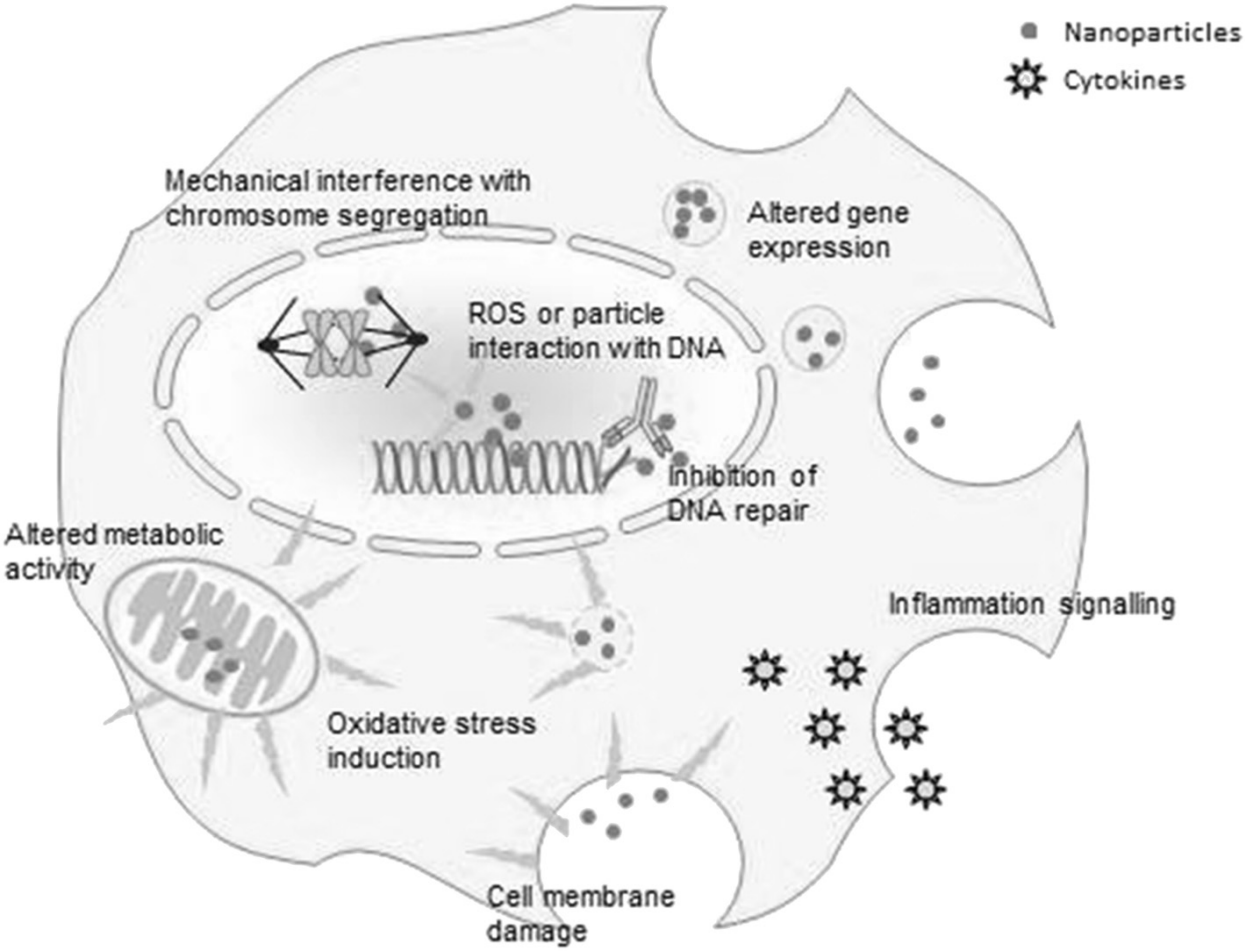
**Suggested mechanisms underlying nanoparticle-induced responses at the cellular level.** At sufficiently high or persistent levels nanoparticle-induced responses potentially lead to altered tissue function and damage. Uptake of nanoparticles by alveolar macrophages can result in the release of mediators and oxidative stress, which may lead to mitochondrial damage, damage to lipids and DNA, and inflammation [104].

The generation of ROS combined with proliferative signals at sites of persistent inflammation may also result in an accumulation of genetic defects. It is therefore important to determine which nanomaterial characteristics determine their persistence in the lung and their ability to cause inflammation. Figure 2 illustrates how nanoparticles can induce adverse effects at the cellular level. It should be noted that some of the mechanisms illustrated in Figure 2 are based on *in vitro* and *(in vivo)* studies that use extremely high and therefore unrealistic concentrations. These results provide evidence for the mechanism behind the observed toxicity. However, they should be evaluated using lower concentrations that resemble realistic inhalation exposure conditions. The mechanistic pathways that operate at low realistic doses might be different from those operating at very high doses when the organism’s defences are overwhelmed [52].

#### Particle size and surface area

Several *in vivo* studies compared the effect of particle size on pulmonary inflammation after inhalation or intratracheal instillation. Ultra-fine anatase titanium dioxide particles of 20 nm induced pulmonary inflammation after intratracheal instillation in rats and mice at lower mass concentrations compared to titanium dioxide particles of 250 nm [10]. Moreover, the onset of the inflammation was earlier in the 20 nm group. However, when the dose was expressed as surface area (measured by the method developed by Brunauer, Emmett, and Teller (BET) [105]), the dose–response curves overlapped [10], indicating that lung inflammation is determined by the total administered surface area. In another study, mice were intratracheally instilled with six types of carbon particles, with primary particle size ranging from 10 to 50 nm and specific surface area ranging from 30 to 800 m^2^/g [36]. Results indicated that particle surface area, measured by BET method, is the best dose metric for responses induced by carbonaceous nanoparticles [36]. In yet another study, however, the total surface area as measured by BET differed from the surface area calculated from particle size, resulting in different dose–response curves. The number of particles was thus considered a better dose metric to describe the effect of carbonaceous nanoparticles after inhalation, and not surface area [106]. These papers show that differences in measuring surface area of nanoparticles and differences in the analysis of experimental data can have a major influence on the results. Still, the studies show a clear effect of particle size in the induction of pulmonary inflammation, regardless if total surface area or particle number is the best dose metric to describe the effect.

Also in other studies, smaller particles caused more severe effects than larger particles when the same mass dose was administered. After intratracheal instillation at a concentration of 0.5 mg/ml, nano-sized nickel oxide particles induced lung inflammation and oxidative stress, but micro-sized nickel oxide particles did not [30]. In addition, several low-toxicity, low-solubility nanoparticles were tested *in vivo* by intratracheal instillation and *in vitro* on a human epithelial cell line. The data showed a clear relation between particle size and pulmonary inflammation as indicated by neutrophil influx and the induction of pro-inflammatory mediators; the smaller the particles, the greater the inflammatory response [107].

To investigate the difference in effect between primary particle size and agglomerate particle size, anatase titanium dioxide particles of diverse primary and agglomerate sizes were tested. When primary particles of three sizes were intratracheally instilled in the lungs of rats at 1.5 mg/kg bw, smaller particles induced greater inflammation in the short-term, but for all groups the inflammation was resolved after one week, regardless of particle size. When anatase titanium dioxide particles with the same primary size but different agglomerate size were tested at 5 mg/kg bw, no clear relationship was observed [31]. When rats were intratracheally instilled with 50 nm primary gold particles, their agglomerate of 200 nm, 250 nm primary gold particles, or their agglomerates of 770 nm, all four groups showed a mild inflammatory reaction at the tested concentration of 1.6 mg/kg bw. No differences were observed between single particles and their agglomerates [28]. However, when rats were exposed for 6 hours by inhalation to titanium dioxide particles with a primary size of 5 nm and an agglomerate size of 30 nm (small agglomerates) or 190 nm (large agglomerates), there was an effect of agglomerate size on pulmonary inflammation [18]. Exposures to both small and large agglomerates at 7 mg/m^3^ resulted in a lung burden of 51.3 and 51.5 μg, respectively and induced increased lactate dehydrogenase (LDH) and oxidative stress markers. Exposure to the large agglomerates significantly increased the number of neutrophils in the lungs, while exposure to the small agglomerates did not [18].

As discussed earlier, the agglomerate size of nanoparticles can change over time. In one study, primary particles of ultra-fine Teflon fume increased in size over time while the particle number decreased, indicating agglomeration. The airborne time allowed ‘aging,’ and after 3.5 minutes of aging, agglomerated Teflon particles exceeded 100 nm and no longer caused toxicity; only freshly generated fumes caused pulmonary inflammation [10].

Particles with a smaller primary size do not always induce more severe effects after inhalation than larger-sized particles in a similar dose. Ferric oxide particles of both 22 nm and 280 nm primary size induced dose-dependent pulmonary inflammation after intratracheal instillation of 0.8 and 20 mg/kg bw, and both sizes induced oxidative stress [37]. Aluminum oxyhydroxide particles of 10 nm and 40 nm primary size and 1.7 μm and 0.6 μm aerodynamic size both induced pulmonary inflammation after 4 weeks inhalation at the highest tested concentration (28 mg/m^3^ exposed concentration, 1100 μg of 10 nm and 1800 μg of 40 nm internal dose in the lungs), with no differences due to particle size [13]. After exposure of mice to 50 μg nano-sized and micro-sized quartz particles by intratracheal instillation, pulmonary inflammation was induced in both groups, without differences due to particle size [33]. Similarly, cerium oxide particles of 5–10 nm (11 mg/m^3^), 40 nm (20 mg/m^3^) and <5000 nm (55 mg/m^3^) primary size induced dose-dependent pulmonary inflammation to the same extent after 28 days of inhalation, perhaps because their aerodynamic particle size was similar, at 1.03 μm 1.17 μm and 1.40 μm, respectively [29].

Overall, the relation between particle size and pulmonary inflammation is not straightforward, suggesting that other parameters also drive the response. With application of similar mass doses of microparticles and nanoparticles, the latter have a higher total surface area and total particle number, which may result in increased pulmonary inflammation. As discussed earlier particles of different sizes differ in patterns of lung deposition and clearance, which influence the actual internal dose that might have an adverse effect on the lungs. For some particles with the same chemical composition and probably within a limited size range, deposited particle surface area seems to be a better predictor for inflammation than exposure concentrations [10,30,31,36,107].

#### Shape

The high aspect ratio of long, thin and rigid carbon nanotubes has raised concern that these carbon nanotubes may induce pulmonary responses similar to asbestos [108-110]. Carbon nanotubes that are of a curly and tangled nature rather than being straight fibres will probably not induce these pulmonary responses. Several studies indicate that multi-walled carbon nanotubes (MWCNT) can induce severe pulmonary inflammation, possibly because of their fibre-shape [41,65,111-114]. One group reported that high-aspect-ratio single-walled carbon nanotubes were 23-fold more inflammatory 1 day after aspiration in mice than an equal mass of spherical carbon black nanoparticles. As stated before, longer rigid fibres cannot be completely taken up by macrophages resulting in frustrated phagocytosis [39,77]. This can lead to an inflammatory response by continuous release of pro-inflammatory mediators, recruitment of inflammatory cells, and generation of reactive oxygen species. In addition, it might disrupt the normal process of motility in the lungs, leading to accumulation of longer fibres in the lower respiratory tract [53,77,115]. In one study, only long nanofibres and long asbestos fibres elicited sustained inflammation in the pleural space, with extensive lesion formation and fibrosis along the parietal pleura [77,116]. There is a cut-off value of 5 μm for long fibres to induce effects in the pleural space [77,117].

Besides fibres, other particle shapes can also influence the toxicity of nanoparticles. After pharyngeal aspiration in mice, at concentrations ranging from 1.88 to 30 μg, anatase titanium dioxide short nanobelts and long nanobelts induced dose- and time-dependent pulmonary inflammation while the nanospheres did not. In addition, there was some accumulation of long nanobelts in the interstitium suggesting increased interstitial access or impaired lymphatic clearance of particles with high aspect ratio [38]. Wire-shaped silver particles induced a strong toxicity at similar particle mass, surface area and number compared to spherical particles on human epithelial cells *in vitro*. In contrast, the various lengths of wire did not affect the level of toxicity [118]. These studies show that fibre-shaped, wire-shaped and nanobelt particles are more toxic to the lungs compared to spherical shaped nanoparticles [38,109,118].

#### Chemical composition

It is plausible that, similar to conventional chemicals, the chemical composition of nanomaterials can influence their effect after inhalation exposure. Nanoparticles consisting of relatively toxic materials such as nickel and cobalt induce severe inflammation, as they have a high surface-specific activity and a large surface area per unit mass [119,120]. Ferric oxide and zinc oxide nanoparticles induced serious hepatic lesions in rats when sprayed directly into the nose twice daily over three days at 8.5 and 2.5 mg/kg bw, respectively. In general, the liver lesions were more severe in animals treated with zinc oxide than those treated with iron oxide. Pulmonary inflammation and lesions were likewise evident in both exposure groups and tended to be more severe in the group exposed to iron oxide [23]. However, for both particle types, effects may have been caused at least in part by dissolved zinc or iron ions. In a long-term inhalation study, rats were exposed for 24 months and mice were exposed for 12 months to similar-sized aerodynamic particles of diesel exhaust at concentrations ranging from 0.8 to 7 mg/m^3^, carbon black at 11.6 mg/m^3^, or titanium dioxide at 10 mg/m^3^[21]. After 24 months exposure, the retained doses in the lungs of rats were 63.9 mg/lung, 43.9 mg/lung and 39.2 mg/lung for the highest exposed concentration of diesel exhaust, carbon black and titanium dioxide, respectively. Compared to controls, the mean lifetime of the rats was substantially shortened by exposure to carbon black and titanium dioxide, but not the diesel exhaust particles. Pulmonary inflammation and lesions were detected in all exposed animals*.* Particles of all three chemical compositions were detected in alveolar macrophages and in the alveolar region. After 6 months and 12 months of exposure, no lung tumours were found in the rats. After 24 months exposure and 6 months recovery, lung tumours were found in rats with all three exposures: 22% for diesel exhaust, 39% for carbon black, and 32% for titanium dioxide particles. It is remarkable that exposure to diesel exhaust resulted in the highest retained dose in the lungs but did not induce the highest tumour rate and did not shorten the lifetime of the rats. In mice, the tumour rate in the exposed groups did not differ from the controls [21].

After short-term inhalation of five days, differences were observed in the effects caused by nanoparticles of seven different chemical compositions [22]. All nanoparticles were tested at concentrations of 0.5, 2.5 and 10 mg/m^3^, except for titanium dioxide nanoparticles that were tested at 2, 10 and 50 mg/m^3^ and multi-walled carbon nanotubes that were tested at 0.1, 0.5 and 2.5 mg/m^3^. Of these, titanium dioxide, cerium dioxide, zinc oxide, and multi-walled carbon nanotubes induced dose-dependent pulmonary inflammation. For the first three, it was reversible at lower concentrations and partly reversible at the highest concentrations tested. The effects of multi-walled carbon nanotubes were irreversible and progressive. Exposure to zirconium dioxide, silicon dioxide, and carbon black induced no detectable inflammation at the tested concentrations. The nanoparticles that caused pulmonary inflammation were retained in the lungs at higher doses compared to the nanoparticles that did not induce pulmonary inflammation. After 5 days inhalation, the retained doses in the lungs were 1635 μg titanium dioxide, 340 μg cerium oxide, 428 μg zinc oxide compared to 200 μg zirconium dioxide, and 93 μg silicon dioxide, for the highest concentrations tested [22]. After intratracheal instillation at a concentration of 0.5 mg/ml, nano-sized nickel oxide particles induced lung inflammation and oxidative stress while nano-sized titanium dioxide particles did not [30]. It must be noted that titanium dioxide nanoparticles occur in different crystal structures: anatase, rutile or a combination of both. Several *in vivo* and *in vitro* studies showed anatase titanium dioxide induced more adverse effects than rutile titanium dioxide [121-125]. According to these studies, chemical composition of the nanoparticles affects their potential to induce pulmonary inflammation, as would be expected from the different potency of conventional chemicals.

#### Surface charge

Nanoparticles have different surface charges depending on the coatings, surfactants, and solvents used in production. In addition, they may acquire a corona of proteins after deposition in the lung. The surface charge can be measured by the zeta-potential, which is the electric potential created between the charged groups associated with the surface of a particle and the suspension medium. The zeta-potential reveals dynamic changes depending on the pH of the medium and the adsorption of proteins that form the corona. In most metal oxide nanoparticles, the zeta-potential is negative in a neutral pH of 7.4, predominantly positive in an acidic environment of pH 5.6, and slightly negative when there is a corona of proteins or lung lining fluid [77]. When nanoparticles encounter biological fluids containing macromolecules, they attract the oppositely charged ones to form the corona. The surface charge will change based on the adsorption of those molecules and proteins, thereby reducing the overall charge of the nanoparticles [78,79]. When they are phagocytized by alveolar macrophages, the proteolytic enzymes and acidic pH in the phagolysosomes may strip off all or part of the corona and reveal the naked surface of the particle, restoring its original zeta-potential [126]. If the zeta-potential has a high positive value, the particle can bind to and damage membranes [77]. Positively charged nanoparticles are more easily taken up by lung cells, compared to neutral or negatively charged nanoparticles; they can thus remain in pulmonary cells for a long time, which may cause severe lung injury [20,127]. When nanoparticles with a high positive zeta-potential interact with the internal face of the lysosomal membrane, lysosomes can be destabilized, triggering cell death and inflammation [128,129]. Similarly, cationic nanoparticles are known to be more cytotoxic *in vitro* than neutral or anionic nanoparticles, causing lysosomal damage [19,130-132]. After intratracheal instillation of 15 metal and metal oxide nanoparticles in mice at a concentration of 150 cm^2^/rat, the ability of the particles to cause acute lung inflammation correlated linearly with their *in vitro* zeta-potential in an acidic environment [19]. For the low-solubility particles, zeta-potential correlated best with the induced pulmonary inflammation [19].

Overall, compared to neutral and negatively charged nanoparticles, positively charged nanoparticles can more easily be taken up by cells [20,127], leading ultimately to cell death and inflammation [128,129].

#### Dissolution in physiological media

Some nanoparticles can dissolve after deposition in the lungs, leading to formation of ions. When fast-dissolving nanoparticles are phagocytized by macrophages, the dissolution rate may be accelerated, leading to lysosomal destabilisation, cell death, and inflammation, dependent on the chemical identity of the ions that are released [84]. For fast-dissolving nanoparticles, the effect is mainly driven by their chemical composition [77]. Copper ions, zinc ions, and silver ions are known to have a toxic effect *in vitro*[42,130]. Mice were intratracheally instilled with a panel of 15 metal or metal oxide nanoparticles to relate their various physicochemical parameters to lung inflammation. Toxic ions, like copper and zinc caused destabilization of the lysosomal membrane [19]. In the acidic conditions of phagolysosomes, nanoparticles of copper oxide, magnesium oxide, and zinc oxide showed rapid, complete dissolution, while nanoparticles like silver, cerium oxide, silica, and titanium dioxide showed minimal dissolution [77]. The pulmonary toxicity of nickel, zinc, and copper oxide nanoparticles and their aqueous extracts (containing only ions and no particles) were investigated both *in vitro* and *in vivo*[43]. Results showed that the pulmonary inflammation induced by nickel oxide nanoparticles is caused by the particles and not by nickel ions in the aqueous extracts. For zinc oxide and copper oxide, the aqueous extracts induced effects similar to their corresponding nanoparticles *in vitro*. However, *in vivo*, zinc oxide and copper oxide nanoparticles caused particle-specific eosinophil recruitment that was not observed after administration of their aqueous extracts. In addition, exposure to the nickel and zinc oxide nanoparticles caused chronic effects that lasted up to four weeks. No aqueous extract caused such sustained inflammation, probably because soluble ions are rapidly cleared from the lungs.

It must be stressed that the dissolution rate of nanoparticles is not a constant factor but depends on particle size, coating, stability, manufacturing process, and biological environment. *In vivo*, released ions may be transported from the site of generation to other body parts, resulting in continued dissolution (and thus ion generation) of the residual nanoparticles. *In vitro*, dissolution may reach a maximum under static conditions. Especially for silver nanoparticles, the literature on their dissolution is contradictory. One study tested agglomeration, sedimentation, and dissolution of silver nanoparticles in biological media, finding that they did not dissolve in any of the tested fluids up to 96 hours incubation [133]. Other researchers report that silver nanoparticles indeed dissolve over time; the smaller the particles the faster they dissolve [134]. Whereas the effect of fast-dissolving nanoparticles probably depends on their chemical composition, the effects of slow- or partial-dissolving nanoparticles are more difficult to predict and will depend on the toxicity of the ions and of the particle that is retained [77].

#### Hydrophobicity

Nanoparticles can be hydrophilic or hydrophobic, based mainly on their surface ligands, surfactants, or stabilizers [130]. Hydrophobic nanoparticles are difficult to disperse in biological fluids and media, while hydrophilic particles easily disperse. However, hydrophobicity enhances the penetration ability of nanoparticles into cell membranes and nuclear pores through the hydrophobic effect [93,135], which is the tendency of nonpolar substances to aggregate in aqueous solution and exclude water molecules [136]. To investigate the difference in effect between hydrophilic and hydrophobic nanoparticles, rats were exposed to three types of synthetic amorphous silica: 12 nm particles of hydrophilic pyrogenic silica, 12 nm particles of hydrophobic silica, and 18 nm particles of hydrophilic precipitated silica. After inhalation exposure for 13 weeks at 1, 6 and 30 mg/m^3^, the most pulmonary inflammation was induced in the group exposed to 12 nm hydrophilic silica, and the least inflammation in the group exposed to 18 nm hydrophilic silica. This is interesting, as both particle types had similar specific surface areas. It must be noted that the 12 nm hydrophilic silica dissolved quickly over time, which might have caused its inflammatory effects and subsequent fast clearance [25]. In a 5-day inhalation study of three types of synthetic amorphous silica at 1, 5 and 25 mg/m^3^, the pyrogenic silica (also known as fumed silica) induced the most pronounced pulmonary inflammation compared to silica gel and precipitated silica. The silica gel induced the least pulmonary inflammation. All three had a similar clearance rate [24]. Like the 13-week study, the 5-day study showed that hydrophilic pyrogenic silica particles induced more severe pulmonary inflammation compared to other forms of silica, indicating that surface hydrophobicity/hydrophilicity can influence the effect of nanomaterials after inhalation. However, other particle characteristics, such as solubility, charge, and aggregation may also play a role.

#### Surface reactivity

Chemical reactions and leakage of constituents occur at the surface of nanoparticles. The number of surface molecules increases exponentially when the particle decreases in diameter. Therefore, nanoparticles have a larger percentage of surface molecules compared to their ‘bulk’ counterparts [52,137]. Surface reactivity is the potency of particles to react with the immediate environment by inducing reactive oxygen species (ROS), leakage of constituents, and other biochemical reactions. It depends on the chemical composition, shape, size, solubility, and surface area of particles [96,138], and is generally determined by measuring the induction of ROS, as pulmonary inflammation is thought to be caused by ROS generation at the nanoparticle surface [93,99-101].

In a 5-day inhalation study, rats were exposed to 20–30 nm titanium dioxide (mixture of 70% anatase and 30% rutile) at a concentration of 88 mg/m^3^, pigmentary 200 nm titanium dioxide (rutile) at a concentration of 274 mg/m^3^, or quartz particles at a concentration of 96 mg/m^3^. The exposure resulted in a retained dose in the lungs of 2025 μg 20–30 nm titanium dioxide, 9182 μg 200 nm titanium dioxide, and 2190 μg quartz at the end of exposure. The two titanium dioxide particles differ in their crystallinity and surface area; the pigmentary titanium dioxide and the quartz particles differ in chemical composition and surface reactivity. All three particle types induced pulmonary inflammation, but it was reversible after 14 days for both types of titanium dioxide while being not reversible for the quartz. The recovery from effects seemed faster for the smaller titanium dioxide nanoparticles compared to the larger ones, which could reflect the higher mass lung burden of the larger particles caused by their higher exposure mass concentration. Overall, the quartz particles induced the most pulmonary inflammation despite their deposited surface area being the smallest. Therefore, the authors conclude that surface reactivity is more important than surface area in nanoparticle toxicity [44]. The same conclusion was reached when ultrafine titanium dioxide particles, differing in specific surface area and in surface reactivity, were tested in rats after intratracheal instillation at 1 and 5 mg/kg bw. Only the titanium dioxide particles with the highest surface reactivity induced pulmonary inflammation [11]. However, those that induced no pulmonary inflammation were reduced in reactivity by a coating of silica or alumina. This finding implies that the rats were exposed to particles with different chemical composition and that toxicity is determined by the composition of the surface that comes in contact with a cell. Similar findings were reported in another study in which various types of quartz particles were intratracheally instilled in rats at 1 and 5 mg/kg bw. The intensity of the resulting pulmonary inflammation was wide-ranging and not dependent on particle size; surface reactivity determined the toxicity of nanoparticles rather than particle size [12]. *In vitro*, quartz particles had a larger inflammatory potential compared to titanium dioxide and carbon black, although the quartz had a lower total surface area. The authors concluded that the greater ability of quartz to cause inflammation is related to its surface oxidative activity. For particles with a highly reactive surface like quartz, lower surface-area doses are required to induce pro-inflammatory responses [139].

Information on the surface reactivity of nanoparticles combines information on the effects of the chemical composition, shape, size, solubility, and surface area of the nanoparticles [96,138]. In addition, several studies found a correlation between surface reactivity and pulmonary inflammation [11,12,44]. Therefore, surface reactivity might be the most important nanoparticle characteristic determining their effect.

#### Methods to determine surface reactivity of nanomaterials

Several methods are available to determine surface reactivity of nanomaterials. As pulmonary inflammation is thought to be caused by the generation of ROS at the nanoparticle surface [93,99-101], this process has been studied in both cell-free and cellular conditions. The oxidation potential of nanoparticles in cell-free conditions can be easily analysed by electron spin resonance (ESR) techniques. These techniques use a spin-trapping agent to detect the nanoparticle-elicited generation of hydroxyl radicals in the presence of hydrogen peroxide. However*,* the process does not mimic the oxidation potential of the particles in the reducing environment of cells or extracellular fluid [96]. One study observed that the acellular potential of 20 nm silver nanoparticles to generate ROS was lower compared to larger silver nanoparticles, whereas its cellular potential was higher [140]. Another study observed that carbon black generated substantial amounts of ROS under cell-free conditions, but titanium dioxide nanoparticles did not. However, both showed a comparable dose-dependent capacity to produce intracellular ROS [141]. These results suggest that the generation of ROS might be an indirect effect of the interaction of the nanoparticles with cellular components. As it occurred in macrophages only at concentrations above those that reduce their metabolic activity, ROS generation may have been a secondary effect rather than causing the onset of cytotoxicity [140]. Therefore, the inability of nanoparticles to produce ROS in cell-free systems does not rule out their potential to produce intracellular oxidative stress [141]. Measuring the intracellular induction of ROS after nanoparticle exposure *in vitro* might be a way to categorize nanoparticles into hazard groups. However, there is no validated *in vitro* assay available to test all types of nanomaterials.

The intracellular induction of ROS can be measured using ESR techniques in combination with *in vitro* cellular exposure or using the 2’-7’-dichlorodihydrofluorecein diacetate (DCFH-DA) assay. The DCFH-DA assay uses a fluorescent probe to visualize the induction of ROS in cells after exposure to nanoparticles. Another method is the free radical analytical system (FRAS) assay that measures the formation of reactive oxygen metabolites (ROM) after exposure to nanoparticles. Besides measuring ROS generation, the surface reactivity of nanomaterials can be measured based on how well they serve the purpose for which they were designed. For example, some nanomaterials are added to a product for their catalytic potential, and others for their anti-bacterial activity or UV absorbance. For such nanomaterials, the surface reactivity required to catalyse reactions, kill bacteria, or absorb UV can be used to determine the actual surface reactivity of the materials. Other methods to measure surface reactivity are the erythrocyte haemolysis assay [142,143] and the vitamin C yellowing assay [11,144]. The erythrocyte haemolysis assay measures the amount of haemoglobin released after exposure of red blood cells to nanoparticles, and the vitamin C assay measures the chemical reactivity of nanoparticles toward an anti-oxidant. Neither of the assays can measure the surface reactivity of all types of nanomaterials.

## Summary and conclusions

Although size has been put forward as an essential parameter to predict the pulmonary inflammation caused by nanomaterials, many other factors modulate the outcomes of toxicity studies. Our conclusions on the physicochemical characteristics of nanomaterials that affect pulmonary inflammation are listed below.

• The induction of pulmonary inflammation by nanomaterials depends largely on the extent of deposition in and clearance from the lungs.

• No single particle property can be identified as the most important in the induction of pulmonary inflammation by nanomaterials, as various properties affect different stages of the events leading to pulmonary inflammation.

• Surface reactivity might be the best predictor for a nanomaterial’s potential to induce pulmonary inflammation.

• There is a lack of information on the potential effects of long-term inhalation exposure to persistent nanomaterials, in terms of a potential delayed onset of pulmonary inflammation and translocation to secondary organs.

One essential step in predicting the risk of adverse human health outcomes based on experimental studies is to elucidate the deposition and clearance of nanomaterials. These processes are largely driven by the physical characteristics of nanoparticles and need to be taken into account when investigating to what extent specific nanomaterial characteristics affect pulmonary inflammation.

Agglomerate particle size and density are the most dominant of all the nanomaterial characteristics that affect lung deposition. Using these values as input, the dose of nanomaterials in the lung can quite accurately be predicted by the Multiple Pathway Particle Dosimetry model (MPPD model). However, it should be realized that other nanomaterial characteristics affecting lung deposition have not been investigated to the same extent, and results of the MPPD model should be interpreted with caution. Primary size, shape, chemical composition, charge, and dissolution rate can all affect clearance rate.

Many physicochemical characteristics of nanomaterials influence the severity of pulmonary inflammation, and no unifying metric can be identified based on the current available evidence. Some studies use rather high and unrealistic exposure concentrations, which might lead to lung particle overload conditions and severe adverse effects in laboratory animals, which will probably not occur under realistic *in vivo* exposure conditions in humans [67]. Results on the influence of primary particle size on pulmonary inflammation after inhalation are rather contradictory. Some studies report increased pulmonary inflammation after exposure to nanoparticles compared to larger micro-sized particles [10,30,31,36,107], while others report no difference [13,37]. As discussed earlier, both the agglomerate size and the primary size of particles affect lung deposition, clearance, and translocation. Therefore, inhalation studies should measure the actual deposition and retention of particles in the lung, preferably as total mass, surface area, and number dose. By comparing the effects of these local doses for the different particle sizes, conclusions can be drawn about the role of size in pulmonary inflammation. For some particles of the same chemical composition (and probably within a limited size range), the deposited surface area seems to be a better predictor for inflammation than mass exposure concentrations [10,30,31,36,107]. In addition, nanoparticles tend to agglomerate or even aggregate, changing particle size and available surface area. Information on the influence of particle agglomeration and aggregation on pulmonary inflammation is very limited.

**Figure 3:**
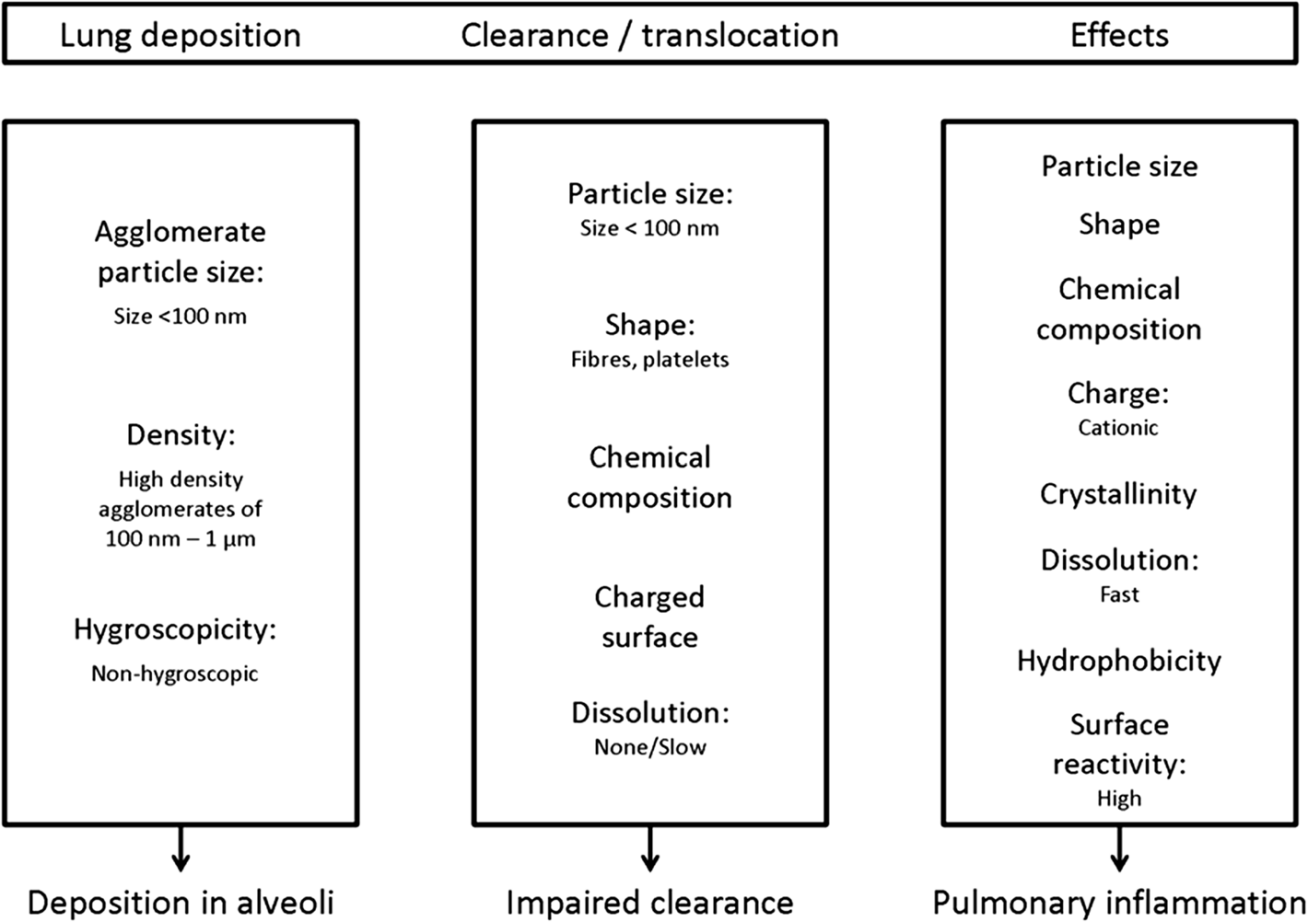
**Nanomaterial characteristics resulting in increased lung deposition (in alveoli), impaired clearance rate, and the induction of pulmonary inflammation.** The figure shows the physico-chemical characteristics of nanoparticles that result in 1) Increased lung deposition in the alveoli (left panel): Nanoparticles with a primary/agglomerate size of <100 nm, or an agglomerate size between 100 nm and 1 μm with a high density, will deposit efficiently in the alveolar region. Non-hygroscopic nanoparticles will not grow in size by water uptake, resulting in a higher chance to reach the alveoli. 2) Impaired clearance rate (middle panel): Particles/agglomerates of <100 nm are less efficiently phagocytised, nano-fibres and –platelets are less efficiently cleared compared to spheres, chemical composition influences clearance rate, charged nanoparticles attract proteins and reduce their clearance, and none or slowly dissolving nanoparticles are less efficiently cleared compared to fast dissolving nanoparticles. 3) The induction of pulmonary inflammation (right panel): After deposition of nanoparticles in the alveoli, the shown characteristics all influence the induction of pulmonary inflammation. Cationic particles are easily taken up by cells, fast dissolving nanoparticles can release toxic ions, and nanoparticles with a high surface reactivity can damage the lungs.

Besides particle size, other nanoparticle characteristics influence deposition, clearance, and induction of pulmonary inflammation (Figure 3). All these characteristics affect different stages of the events leading to pulmonary inflammation; no single characteristic can be identified as the most important in the induction of pulmonary inflammation by nanomaterials.

Measuring the surface reactivity of nanoparticles might be the best way to predict the toxicity of nanoparticles because it combines information on the effects of the chemical composition, shape, size, dissolution rate, and surface area of the particles [96,138]. A nanoparticle’s surface reactivity depends on the exposure medium, in which proteins or other macromolecules might attach to the surface of the nanoparticles. As nanomaterials can differ in all physicochemical characteristics, surface reactivity should be measured separately for each type of nanomaterial in the appropriate medium. Since there is no validated *in vitro* assay available to test all types of nanomaterials, a high-throughput *in vitro* assay should be developed and validated to determine the intracellular production of ROS after nanoparticle exposure. Still, animal studies are needed to validate the assays, as surface reactivity *in vitro* may differ from surface reactivity *in vivo*, when nanoparticles react with macromolecules in the body and where cellular concentrations are much lower.

If surface reactivity indeed turns out to be the best predictor for the toxicity of nanomaterials, it could be used to categorize nanomaterials into hazard groups. This might help reduce animal testing and speed up risk-assessment procedures, which are currently approached on a case-by-case basis. In addition, if a universal unit of surface reactivity can be defined, it can be included in the dose metric of nanomaterials. Still, while surface reactivity and the ability of nanomaterials to produce oxidative stress appear to be important predictors for toxicity in the lung, the effects of other nanomaterial characteristics should not be ruled out completely. Not all nanomaterial characteristics have been investigated to the same extent, and new types of nanomaterials are continuously being developed, taking on more complex forms that may be associated with new mechanisms of toxicity. A close collaboration between developers of nanomaterials and nanotoxicologists is necessary for the development of new nanomaterials with promising benefits and low risks.

Although very little is known on the exact clearance rates and retention of nanoparticles in the lungs, there is concern that non-soluble nanoparticles will be retained in the lungs and secondary organs for years. The phagocytosis of nanoparticles by macrophages is slower compared to microparticles [69,70,72], and nanoparticles deposit deeper in the lungs, where the clearance is slower due to increased pathway length and decreased mucous velocity [59]. A low-level but longer-lasting or repeated exposure might enable delayed or slowly developing pulmonary inflammation that is not resolved over time. Compared to larger particles, increased retention of nanoparticles may damage the lungs or may result in the translocation to secondary organs. It is important to note that in general, translocation to the systemic circulation is very low, below 0.5% of the exposure concentration [16,17,27,34]. It is not clear if there is a cut-off point in particle size beyond which particles can no longer translocate; under overload conditions, even larger particles might translocate.

Long-term animal studies are very expensive; therefore, there is a great need for alternative methods or new ways of combining information to predict long-term accumulation and effects of nanomaterials in the body. Even for short-term effects, not all characteristics can be studied in detail in animal studies, because the variation in nanomaterials is just too large. Therefore, alternative methods need to be developed for high-throughput screening of nanomaterials*.* Some co-culture *in vitro* systems have been developed that mimic the lung barrier [145-149] and hold promise for testing nanomaterials with diverse characteristics, although parallel *in vivo* studies to demonstrate their predictive value are often lacking. Finally, human epidemiological studies conducted in the workplace, for example, can provide useful information on the effects of nanomaterials after inhalation.

Since no single nanoparticle property can be identified as the most important in the induction of pulmonary inflammation, we have some recommendations for future research listed below. The ultimate goal is to categorize nanomaterials according to their characteristics, which would be of immense value for the risk assessment of these wide-ranging and fast-developing products.

• The impact of a nanomaterial characteristic should be systematically tested in different experimental setups by using a number of different nanomaterials, varying only in a single physicochemical characteristic e.g. surface reactivity, and analysed by controlling for other variables such as size and chemistry.

• Surface reactivity should be investigated as a valuable predictor for a nanomaterial’s potential to induce pulmonary inflammation.

• The data from studies that systematically tested a single nanomaterial characteristic can be combined into a large data set to carry out multivariate analysis to determine potential combinations of characteristics that are important in affecting pulmonary inflammation.

• Agglomerate particle size should be measured as closely as possible to the site of exposure in animal studies or in occupational exposure settings in order to accurately predict the lung deposition by the particle dosimetry models such as the MPPD model.

• Nanomaterial characteristics should be measured in the appropriate medium, because reaction with macromolecules in biological fluids will change nanoparticle characteristics and thereby their cellular uptake and effect.

• There is a need to generate data on biodistribution and accumulation of nanomaterials upon long-term exposure, as well as the induction of toxic effects, to assess which of the physicochemical properties have the largest influence on delayed or chronic pulmonary inflammation.

## Competing interests

The authors declare that they have no competing interests.

## Authors’ contributions

HB drafted the manuscript. MP and FRC contributed to the concept and design of the manuscript. MP, FRC, IG and WHdeJ have been involved in revising the manuscript critically for important intellectual content. All authors read and approved the final manuscript.
